# Supplementary material for: Hierarchical and scaffolded phosphorylation of two degrons controls PER2 stability
Source: J Biol Chem. 2024 May 20;300(6):107391. doi: 10.1016/j.jbc.2024.107391 (PMC11223080; doi:10.1016/j.jbc.2024.107391)
Supplement: Supporting Information [file mmc1.pdf]

Supplemental Figures

Supp Fig 1A

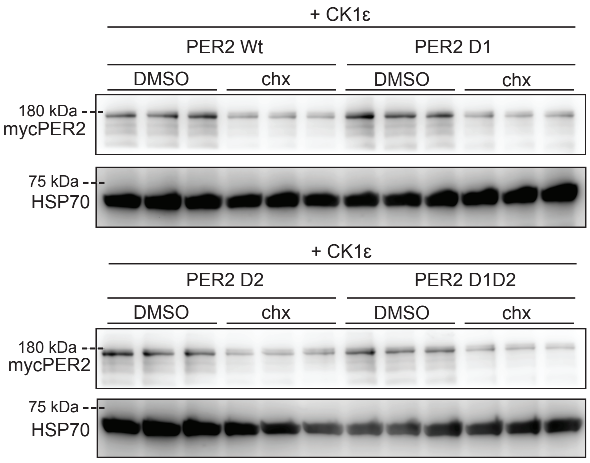

Supp Fig 1B

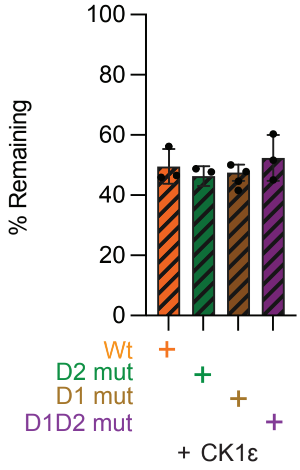

A) Immunoblot of non-luciferase fused PER2's recapitulate results of BDA. PER2 and CK1ε were transiently co-expressed as above. Samples were harvested 4 hours after chx or DMSO addition, in triplicates. PER2 abundance was assessed with SDS-PAGE and Immunoblot.

B) Intensity of PER2 bands from Supp. 1A was quantified and % remaining PER2 was calculated relative to DMSO treated samples. Points represent individual samples, with error bars indicating ±SD.

Supplemental Figure 2

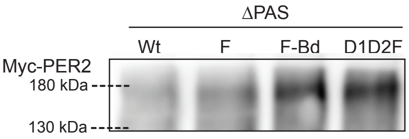

Expression of PER2::luc ΔPAS mutants in HEK293 cells. All constructs expressed measurable luciferase activity as shown in Figure 4.

Supp Fig 3A

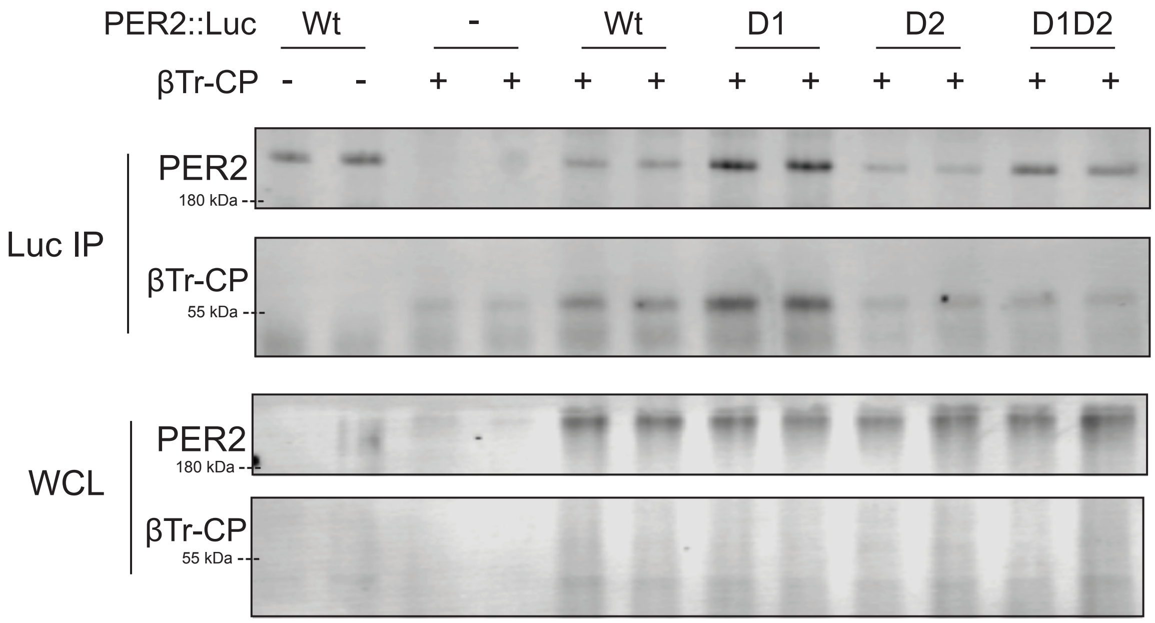

Supp Fig 3B

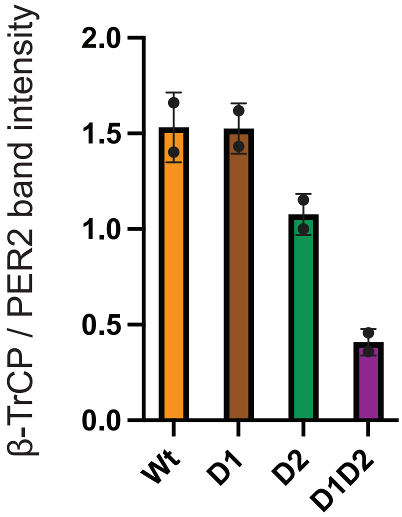

A) D1D2 degron mutant further reduces B-TrCP interaction compared to D2 mutant. β-TrCP-binding was assessed by immunoprecipitation of PER2 with luciferase before SDS-PAGE and immunoblot for myc-β-TrCP. WCL = whole cell lysate.

B) Intensity of β-TrCP IP bands from Supp. 3A was normalized against PER2 IP bands then quantified. Points represent individual samples, with error bars indicating ±SD.
